# Associations among inflammation, mental health, and quality of life in adults with metabolic syndrome

**DOI:** 10.1186/s13098-018-0367-9

**Published:** 2018-08-31

**Authors:** Ji-Ryang Kim, Ha-Na Kim, Sang-Wook Song

**Affiliations:** Department of Family Medicine, St. Vincent’s Hospital, College of Medicine, The Catholic University of Korea, Jungbu-daero 93, Paldal-gu, Suwon-si, Gyeonggi-do 16247 Republic of Korea

**Keywords:** Metabolic syndrome, Inflammation, Mental health, Quality of life

## Abstract

**Background:**

Metabolic syndrome (MetS), a pro-inflammatory state, has become increasingly common worldwide and is a major risk factor for type 2 diabetes mellitus and cardiovascular disease. Recently, studies on the relationships among inflammation, mental health, quality of life, and other diseases have been conducted.

**Methods:**

We investigated the relationship between serum high-sensitivity C-reactive protein (hs-CRP) levels, as an indicator of inflammation, and the quality of life and psychiatric symptoms of Korean adults with MetS. The analysis used data from the Korean National Health and Nutrition Examination Survey, a cross-sectional survey of Korean civilians conducted from January to December 2015. Data from 1600 participants were analyzed in this study. Quality of life was assessed using the EuroQol 5-dimension (EQ-5D) instrument.

**Results:**

Serum hs-CRP levels showed a significant inverse correlation with the EQ-5D index of the overall quality of life. High serum hs-CRP levels were positively associated with mobility problems and suicidal ideation in adults with MetS (multivariate-adjusted odds ratio [OR] 1.66, 95% confidence interval [CI] 1.03–2.66, *p* = 0.036; and multivariate-adjusted OR 2.48, 95% CI 1.23–4.99, *p* = 0.011).

**Conclusions:**

These findings suggest that the elevated inflammatory status in MetS is associated with decreased quality of life and mental health problems. Further prospective studies are needed to confirm the impact of inflammation on the quality of life and mental health of adults with MetS.

## Background

Metabolic syndrome (MetS), which is a common major health issue throughout the world [1], is a multiplex risk factor for atherosclerotic cardiovascular disease and type 2 diabetes [2, 3]. MetS consists of five components: abdominal obesity, elevated serum triglycerides and glucose, increased blood pressure, and reduced levels of high-density lipoprotein (HDL) cholesterol. The presence of three or more of these components constitutes a clinical diagnosis.

The association between MetS and systemic inflammation has been increasingly recognized [4]. Excess energy intake and obesity are known to be associated with low-grade, systemic inflammation [5]. Under these conditions, metabolic cells coordinate the systemic inflammatory process. Excess adipocytes release a variety of proinflammatory cytokines, such as interleukin-6 (IL-6), tumor necrosis factor (TNF), and monocyte chemoattractant protein-1, which mobilize macrophages to adipose tissue to further stimulate cytokine release. These inflammatory conditions may induce insulin resistance in skeletal muscle, leading to blood glucose disorders [6], and may induce macrophage differentiation, oxidation of low-density cholesterol, and lipid-laden foam formation in the arterial lining, resulting in atherosclerosis. Thus, inflammation is associated with an increased likelihood of developing an acute cardiovascular event [7, 8]. Additionally, these conditions can alter the hypothalamus–pituitary–adrenal (HPA) axis [9].

Inflammation is known to be associated with mental health [9–20] and quality of life [21, 22]. Psychological conditions are related to the endocrine and immune systems, especially inflammation and the regulation of the HPA axis. Stress promotes the inflammatory response and affects the HPA axis, leading to anti-inflammatory regulation [12, 18, 23]. There have been many studies on the relationship between depression and inflammation, and the relationship is thought to be complex and interactive [14, 15, 17, 19]. In particular, depression associated with increased inflammation is correlated with suicidal ideation and attempts [10, 11, 18, 24]. Additionally, inflammation may reduce quality of life as a result of reduced happiness [21]. Therefore, the management of inflammation is critical for maintaining mental health and quality of life.

C-reactive protein (CRP) is synthesized by the liver in response to several inflammatory factors, such as IL-6 and TNF-α, and is used mainly as a biomarker of systemic inflammation. High-sensitivity (hs)-CRP is a more sensitive test for subtle inflammation, and serum hs-CRP levels may reflect the inflammatory state of various diseases. There have been many studies evaluating disease risk using serum hs-CRP, and several studies using serum hs-CRP have shown that cardiovascular disease and diabetes are positively correlated with systemic inflammation during disease progression [25–27]. Elevated serum CRP levels have been associated with obesity and systemic inflammation in MetS [4]. Additionally, the association between inflammation, as indicated by serum CRP levels, and psychiatric disorders has been studied [16, 24, 28, 29], and some studies using serum CRP have shown that chronic inflammation may exert a significant negative impact on quality of life [21, 22].

We evaluated whether serum hs-CRP levels are associated with health-related quality of life and psychiatric symptoms in adults with MetS using data from the Korean National Health and Nutrition Examination Survey (KNHANES).

## Methods

### Study population

We used data collected by the KNHANES VI-3, which was conducted from January to December 2015. The KNHANES is implemented by the Korea Center for Disease Control and Prevention (KCDC) over 3-year intervals to assess the status of public health and to provide baseline data for the development, establishment, and evaluation of public health policies in the Korean population. KNHANES participants comprise non-institutionalized individuals ≥ 1 year of age selected using a stratified, multi-stage cluster probability sampling design to ensure an independent, homogeneous, and nationally representative sample. Data are collected by a variety of means, including household interviews, anthropometric and biochemical measurements, and nutritional status assessments [30]. All protocols were approved by the Institutional Review Board of the KCDC, and participants provided written informed consent at baseline. In the KNHANES VI-3, 7380 participants completed the survey. In this cross-sectional study, we initially examined data from 5855 adults 20 years of age or older. We excluded those participants with missing information or values for major variables (n = 72) and those without MetS (n = 4183). Thus, the final sample of the present study included 1600 participants. This study was approved by the Institutional Review Board of the Catholic University of Korea (IRB Approval Number: VC18ZESI0030).

### Definitions of variables

We used the revised criteria of the National Cholesterol Education Program Adult Treatment Panel III (NCEP-ATP III) to define MetS [31]. The NCEP-ATP III criteria define MetS as the presence of any three or more of the following five MetS components: waist circumference ≥ 90 cm (≥ 85 cm for women) according to the Korean Society for the Study of Obesity cut-off point for abdominal obesity [32]; triglyceride levels ≥ 150 mg/dL or taking medication for elevated triglycerides; HDL cholesterol levels < 40 mg/dL (< 50 mg/dL for women) or taking medication to reduce HDL-cholesterol; systolic blood pressure ≥ 130 mmHg or diastolic blood pressure ≥ 85 mmHg or taking antihypertensive medication; and fasting glucose levels ≥ 100 mg/dL or taking medication for elevated glucose levels.

We obtained information from a survey on four psychiatric symptoms. Sleep was classified into two categories: proper sleep (slept more than 7 h) and improper sleep (slept less than 7 h). Participants were divided into the following two groups according to how much stress they felt in their daily lives: those not feeling stress and those feeling stress. Depressed mood was defined as an affirmative response to “Have you ever felt so sad or hopeless that you experienced difficulties in daily life on a continuous basis for more than 2 weeks during the past year?” We also asked participants if they had ever felt like committing suicide during the past year.

The EuroQol 5-dimension (EQ-5D) instrument is a self-administered patient questionnaire that is the most widely used generic preference-based measure of health-related quality of life. EQ-5D respondents classify their own health status into five dimensions (mobility, self-care, usual activities, pain/discomfort, and anxiety/depression) with three levels of severity (no problems, some problems, or extreme problems). A unique health state is defined by combining one level from each of the five dimensions. A total of 243 possible health states can be defined in this way. EQ-5D health states, defined by the EQ-5D descriptive system, can be converted into a single index, the EQ-5D index, by applying a formula that essentially attaches weights to each of the levels in each dimension. In this study, we employed the weights that were developed by the KCDC in 2007 in consideration of the characteristics of Koreans; the index ranged from the most imperfect health status, − 0.171, to the most perfect health status as + 1 [30].

### Laboratory measurements

Blood samples were collected from the antecubital vein of each participant after at least 12 h of fasting; they were then processed, refrigerated immediately, and transported in cold storage to the Central Testing Institute in Seoul, Korea. All blood samples were analyzed within 24 h of arrival at the testing facility. Fasting plasma glucose, triglycerides, HDL cholesterol, and creatinine levels were measured using an auto-analyzer (Hitachi Automatic Analyzer 7600; Hitachi Ltd., Tokyo, Japan).

Serum hs-CRP levels were determined using an immunoturbidimetric assay (Cobas; Roche Diagnostics, Basel, Switzerland). Serum hs-CRP levels were categorized by quartiles with quartile 1 (Q1) representing the lowest CRP levels; Q2 representing low-medium CRP levels; Q3 representing high-medium CRP levels; and Q4 representing the highest CRP levels.

### Clinical and anthropometric measurements

The anthropometric measurements of the participants were taken by specially trained examiners. Height and weight were measured after an overnight fast while the participants wore lightweight gowns, and waist circumference was measured using a measuring tape on the horizontal plane around the umbilical region after the subject exhaled. Blood pressure measurements were taken with subjects in the seated position after a rest period of at least 5 min. Body mass index (BMI) was calculated as each participant’s weight (in kilograms) divided by the square of height (in meters). Obesity was defined as a BMI ≥ 25.0 kg/m^2^ [33].

Self-reported information regarding age, sex, household income, smoking, alcohol consumption, amount of physical activity, and past or current medical problems was obtained. Cigarette smoking status was divided into three categories based on current use estimates: non-smoker, ex-smoker, and current smoker. Alcohol consumption was classified into three categories: abstinence (no alcoholic drinks consumed within the last year), moderate drinking (fewer than 14 standard drinks consumed per week for men or seven for women), and heavy drinking (more than 14 standard drinks consumed per week for men or more than seven for women). Physical activity was classified as low or not low. Low physical activity was defined as 150 min or less of moderately intense exercise per week or 75 min or less of highly intense exercise per week. Household income was classified using monthly equivalized household income (quartiles), which was estimated as the total monthly household income divided by the square root of the total number of household members.

### Statistical analysis

To analyze the data, which were collected using a complex sampling design, we applied the SAS PROC SURVEY module with consideration accorded to strata, clusters, and weights. All analyses were performed using the sample weights from the KNHANES. The characteristics of the study population according to serum hs-CRP quartiles were analyzed using independent *t*-tests for continuous variables and Chi square tests for dichotomous variables. The data are expressed as means ± standard errors or percentages. We used multiple logistic regression analysis to examine the associations of psychiatric symptoms and quality of life, treated as dependent variables, with serum hs-CRP levels, treated as the independent variable. Model 1 was adjusted for age and sex, and Model 2 was adjusted for age, sex, income, smoking status, alcohol consumption, physical activity, BMI, and history of comorbidities, including dyslipidemia, diabetes mellitus, hypertension, and other infectious or inflammatory diseases, such as sinusitis, otitis media, atopic dermatitis, or rheumatoid arthritis. The correlation between serum hs-CRP levels and EQ-5D indices was analyzed using simple linear regression analysis.

## Results

### Characteristics of participants

Table 1 shows the characteristics of study participants according to serum hs-CRP quartiles. Significant differences in smoking, obesity, waist circumference, fasting glucose, and HDL cholesterol were observed according to serum hs-CRP level (Table 1).

**Table 1:** Characteristics of the study participants according to serum hs-CRP level quartile

	Q1 (n = 359)	Q2 (n = 410)	Q3 (n = 421)	Q4 (n = 410)	p value
Serum hs-CRP (mg/L)	0.30 ± 0.01	0.59 ± 0.01	1.06 ± 0.01	4.28 ± 0.20	–
Age	56.3 ± 0.9	57.0 ± 0.9	54.6 ± 0.9	53.4 ± 1.0	*0.013*
Sex (male)	47.1	152.1	51.0	55.7	0.291
Low household income	22.6	20.8	21.9	25.2	0.275
Heavy drinking	22.3	19.1	20.1	19.3	0.889
Current smoking	29.3	24.1	24.3	25.4	*0.010*
Low physical activity	53.4	52.4	56.9	60.4	0.253
Obesity	52.9	62.7	67.7	74.5	*< 0.001*
Waist circumference (cm)	87.4 ± 0.5	89.8 ± 0.4	90.8 ± 0.5	92.9 ± 0.7	*< 0.001*
SBP (mmHg)	127.3 ± 1.0	127.7 ± 0.9	126.5 ± 0.9	128.7 ± 0.9	0.347
DBP (mmHg)	79.9 ± 0.7	79.3 ± 0.6	79.7 ± 0.6	80.3 ± 0.6	0.604
Fasting glucose (mg/dL)	108.6 ± 1.4	110.5 ± 1.8	112.4 ± 1.6	116.5 ± 2.1	*0.009*
Triglycerides (mg/dL)	190.5 ± 8.8	219.9 ± 10.5	206.3 ± 8.3	218.6 ± 11.7	0.092
HDL-cholesterol (mg/dL)	45.9 ± 0.7	44.2 ± 0.6	43.1 ± 0.6	41.4 ± 0.6	*< 0.001*
Comorbidities					
Diabetes	16.6	14.6	16.3	15.7	0.920
Dyslipidemia	33.5	34.0	23.1	20.8	*< 0.001*
Hypertension	44.3	44.3	41.1	42.2	0.812

Values are expressed as means ± standard errors or percentages. Results in italics indicate statistical significance at the 0.05 level. Quartile 1 (Q1): the lowest hs-CRP levels; Q2: low-medium hs-CRP levels; Q3: high-medium hs-CRP levels; and Q4: the highest hs-CRP levels

*Hs-CRP* high-sensitivity C-reactive protein, *SBP* systolic blood pressure, *DBP* diastolic blood pressure, *HDL* high-density lipoprotein

### Correlation between hs-CRP levels and EQ-5D index

**Fig. 1:**
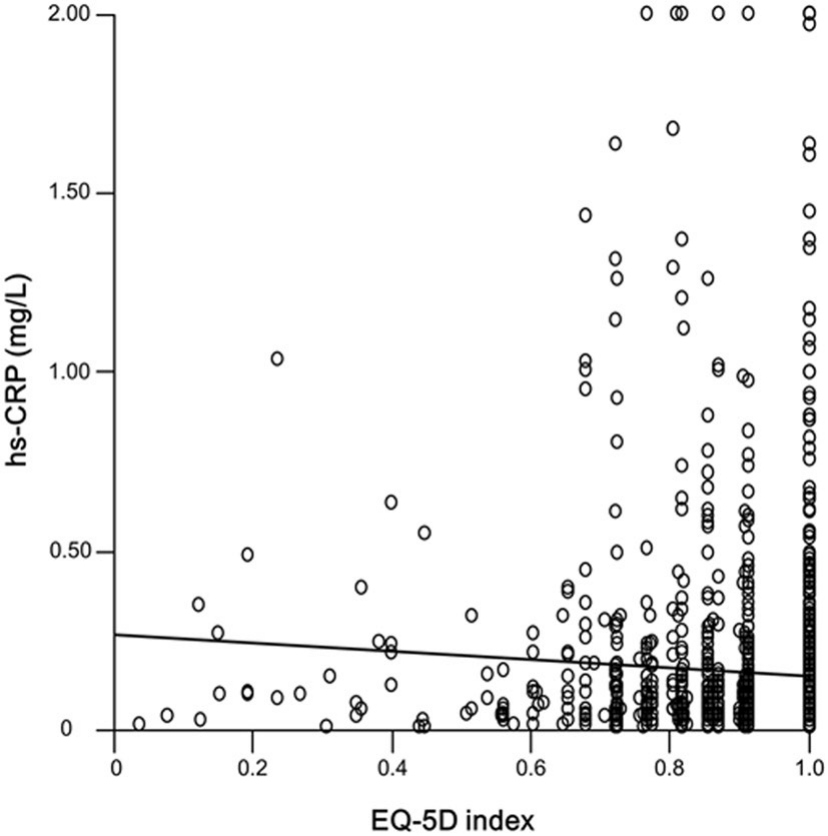
Correlation between high-sensitivity C-reactive levels and EuroQol 5-dimension index

The EQ-5D index showed a significant inverse correlation with serum hs-CRP levels (*R*^2^ = 0.0006, B = − 1.64, *p *= 0.018; Fig. 1).

### Association of quality-of-life problems and psychiatric symptoms with serum hs-CRP levels

Relationships between the frequency of problems in the five dimensions of the EQ-5D and serum hs-CRP levels are shown in Table 2. No differences were observed in the frequency of quality-of-life problems by serum hs-CRP quartile.

**Table 2:** The frequency of problems in quality of life according to serum hs-CRP level quartile

	Q1	Q2	Q3	Q4	p	p for trend
Mobility	17.8	20.5	17.0	22.1	0.311	0.381
Self-care	3.9	3.9	5.0	6.3	0.384	0.118
Usual activities	9.6	12.4	9.4	12.6	0.351	0.532
Pain/discomfort	27.2	29.1	22.9	31.4	0.077	0.602
Anxiety/depression	14.5	14.5	8.9	11.4	0.064	0.085

Values are expressed as percentages. Quartile 1 (Q1): the lowest hs-CRP levels; Q2: low-medium hs-CRP levels; Q3: high-medium hs-CRP levels; and Q4: the highest hs-CRP levels

*Hs-CRP* high-sensitivity C-reactive protein

Relationships between the four psychiatric symptoms and the serum hs-CRP levels are shown in Table 3. As serum hs-CRP levels increased, suicidal ideation increased in adults with MetS (*p* for trend = 0.002). However, no significant differences were observed for the other psychiatric symptoms according to serum hs-CRP quartile.

**Table 3:** The frequency of psychiatric symptoms according to serum hs-CRP level quartile

	Q1	Q2	Q3	Q4	p-value	p for trend
Stress	29.3	26.8	29.9	29.5	0.842	0.718
Depressed mood	16.2	11.0	12.5	13.4	0.299	0.515
Suicide ideation	4.1	3.0	5.9	9.2	*0.002*	*0.002*
Improper sleep	54.1	52.2	47.7	54.9	0.261	0.922

Values are expressed as percentages. Results in italics indicate statistical significance at the 0.05 level. Quartile 1 (Q1): the lowest hs-CRP levels; Q2: low-medium hs-CRP levels; Q3: high-medium hs-CRP levels; and Q4: the highest hs-CRP levels

*hs-CRP* high-sensitivity C-reactive protein

The unadjusted odds ratios (ORs), age- and sex-adjusted ORs (Model 1), and multivariate-adjusted ORs (Model 2) for the five dimensions of the EQ-5D and the four psychiatric symptoms are shown in Tables 4 and 5 according to serum hs-CRP level. Mobility problems were associated with high serum hs-CRP levels in adults with MetS (age- and sex-adjusted OR 1.73, 95% confidence interval [CI] 1.10–2.72, *p *= 0.018; multivariate-adjusted OR 1.66, 95% CI 1.03–2.66, *p *= 0.036). However, no associations between self-care, usual activities, pain/discomfort, or anxiety/depression and serum hs-CRP levels were detected in adults with MetS. Among the psychiatric symptoms, suicidal ideation was positively associated with serum hs-CRP levels in adults with MetS (unadjusted OR 2.36, 95% CI 1.22–4.57, *p* = 0.011; age- and sex-adjusted OR 2.61, 95% CI 1.35–5.04, *p* = 0.005; multivariate-adjusted OR 2.48, 95% CI 1.23–4.99, *p *= 0.011). However, no associations were detected between stress, depressed mood, or sleep duration and serum hs-CRP level in adults with MetS.

**Table 4:** Odds ratios and 95% confidence intervals of problems in quality of life according to serum hs-CRP level quartile

	Crude		Model 1		Model 2	
	OR (95% CI)	p	OR (95% CI)	p	OR (95% CI)	p
Mobility						
Q1	1		1		1	
Q2	1.19 (0.76–1.86)	0.435	1.18 (0.74–1.89)	0.489	1.16 (0.73–1.85)	0.529
Q3	0.94 (0.63–1.41)	0.779	1.04 (0.68–1.61)	0.855	0.99 (0.63–1.56)	0.965
Q4	1.31 (0.84–2.04)	0.225	1.73 (1.10–2.72)	*0.018*	1.66 (1.03–2.66)	*0.036*
Self-care						
Q1	1		1		1	
Q2	1.00 (0.48–2.07)	0.992	0.96 (0.46–1.99)	0.911	0.97 (0.46–2.04)	0.933
Q3	1.31 (0.60–2.85)	0.498	1.42 (0.64–3.15)	0.389	1.43 (0.61–3.33)	0.410
Q4	1.67 (0.80–3.47)	0.169	1.92 (0.92–4.00)	0.083	2.00 (0.91–4.39)	0.085
Usual activities						
Q1	1		1		1	
Q2	1.34 (0.81–2.19)	0.250	1.33 (0.81–2.19)	0.260	1.33 (0.81–2.19)	0.263
Q3	0.97 (0.58–1.63)	0.914	1.04 (0.62–1.77)	0.873	1.00 (0.58–1.74)	0.999
Q4	1.35 (0.79–2.31)	0.264	1.61 (0.95–2.72)	0.074	1.59 (0.89–2.83)	0.114
Pain/discomfort						
Q1	1		1		1	
Q2	1.10 (0.76–1.59)	0.611	1.12 (0.78–1.60)	0.549	1.04 (0.72–1.50)	0.850
Q3	0.80 (0.56–1.12)	0.189	0.83 (0.59–1.16)	0.276	0.77 (0.54–1.11)	0.155
Q4	1.23 (0.84–1.81)	0.294	1.38 (0.95–2.01)	0.087	1.38 (0.93–2.02)	0.106
Anxiety/depression						
Q1	1		1		1	
Q2	1.00 (0.65–1.55)	0.990	1.02 (0.66–1.58)	0.919	1.05 (0.68–1.64)	0.817
Q3	0.58 (0.36–0.93)	0.025	0.60 (0.38–0.96)	0.032	0.61 (0.38–1.00)	0.052
Q4	0.76 (0.48–1.20)	0.235	0.83 (0.53–1.32)	0.435	0.92 (0.56–1.53)	0.757

Quartile 1 (Q1): the lowest hs-CRP levels; Q2: low-medium hs-CRP levels; Q3: high-medium hs-CRP levels: and Q4: the highest hs-CRP levels. Model 1: adjustment for age and sex, Model 2: adjustment for age, sex, income, smoking, alcohol consumption, physical activity, BMI, and history of comorbidities including dyslipidemia, diabetes mellitus, hypertension, and other infectious or inflammatory diseases. Results in italics indicate statistical significance at the 0.05 level

*hs-CRP* high-sensitivity C-reactive protein, *OR* odds ratio, *CI* confidence interval

**Table 5:** Odds ratios and 95% confidence intervals of psychiatric symptoms according to hs-CRP tertiles

	Crude		Model 1		Model 2	
	OR (95% CI)	p	OR (95% CI)	p	OR (95% CI)	p
Stress						
Q1	1		1		1	
Q2	0.88 (0.61–1.27)	0.505	0.91 (0.63–1.32)	0.610	0.85 (0.59–1.24)	0.402
Q3	1.03 (0.71–1.50)	0.879	0.97 (0.66–1.44)	0.894	0.92 (0.62–1.36)	0.670
Q4	1.01 (0.69–1.48)	0.943	0.91 (0.61–1.37)	0.659	0.86 (0.56–1.30)	0.467
Depressed mood						
Q1	1		1		1	
Q2	0.64 (0.39–1.04)	0.068	0.64 (0.39–1.04)	0.072	0.62 (0.38–1.02)	0.061
Q3	0.74 (0.46–1.20)	0.220	0.77 (0.48–1.25)	0.291	0.73 (0.45–1.18)	0.194
Q4	0.80 (0.51–1.24)	0.311	0.89 (0.58–1.38)	0.604	0.86 (0.55–1.35)	0.509
Suicide ideation						
Q1	1		1		1	
Q2	0.73 (0.35–1.50)	0.388	0.74 (0.36–1.52)	0.412	0.72 (0.34–1.50)	0.375
Q3	1.46 (0.69–3.08)	0.325	1.52 (0.72–3.20)	0.271	1.30 (0.59–2.83)	0.516
Q4	2.36 (1.22–4.57)	*0.011*	2.61 (1.35–5.04)	*0.005*	2.48 (1.23–4.99)	*0.011*
Improper sleep						
Q1	1		1		1	
Q2	0.93 (0.67–1.29)	0.658	0.92 (0.66–1.28)	0.615	0.96 (0.69–1.32)	0.790
Q3	0.77 (0.58–1.04)	0.087	0.77 (0.57–1.03)	0.072	0.81 (0.60–1.09)	0.164
Q4	1.04 (0.74–1.45)	0.841	1.01 (0.73–1.40)	0.945	1.06 (0.76–1.48)	0.721

Quartile 1 (Q1): the lowest hs-CRP levels; Q2: low-medium hs-CRP levels; Q3: high-medium hs-CRP levels; and Q4: the highest hs-CRP levels. Model 1: adjustment for age and sex, Model 2: adjustment for age, sex, income, smoking, alcohol consumption, physical activity, BMI, and history of comorbidities including dyslipidemia, diabetes mellitus, hypertension, and other infectious or inflammatory diseases. Results in italics indicate statistical significance at the 0.05 level

*hs-CRP* high-sensitivity C-reactive protein, *OR* odds ratio, *CI* confidence interval

## Discussion

MetS is a major risk factor for cardiovascular disease and type 2 diabetes mellitus, diseases with high mortality and morbidity worldwide. It has been determined that a pro-inflammatory state is one of the mechanisms underlying this pathological condition. In recent years, research on the relationship between inflammation and mental health has continued, and studies have been conducted on the relationships among major diseases (e.g., cardiovascular disease), inflammation, and mental health. In this study, we investigated whether serum hs-CRP levels, an indicator of inflammation, are associated with health-related quality of life and psychiatric symptoms in Korean adults with MetS. These relationships are very complex and reciprocal, and the cross-sectional nature of our observations prevents us from making causal considerations between the variables studied. We found that high serum hs-CRP levels in adults with MetS were associated with mobility problems and suicidal ideation. The EQ-5D index, an assessment of overall quality of life, showed a significant inverse correlation with serum hs-CRP levels.

A number of studies have reported associations between multiple chronic diseases and quality of life, and MetS is also known to reduce quality of life [34, 35]. In other studies, chronic inflammation has exhibited a negative relationship with quality of life [21] and a positive relationship with pain and disability-related problems [22]. In a cross-sectional study of depressed patients, high CRP levels were associated with somatic symptoms [36]. In this study, high serum hs-CRP levels were associated with poor overall quality of life and mobility problems. This can be explained by the sickness behavior model of sickness syndrome theory [16, 37]. Inflammation can cause symptoms by directly affecting the central nervous system, and CRP is a common marker of such inflammation. These symptoms are known to negatively impact quality of life.

MetS are known to associate with low mental health [38–42]. The relationship between MetS and low mental health might be mediated through indirect effects on health behavior as well as direct effects on the stress system, including the HPA axis, the autonomic nervous system, and immune system [17, 43, 44]. Cross-sectional [39, 45] and longitudinal studies [38, 46] have shown higher work stress is associated with higher risk of MetS. In a study about associations among obstructive sleep apnea syndrome, MetS, and mental health [40], early-stage obstructive sleep apnea was associated with worsening of psychological conditions. Depression and anxiety are also known to associate with inflammation and MetS in recent studies [12–17, 20, 29, 40, 41, 44, 47–50]. We didn’t observe any association between serum hs-CRP levels and stress, depressed mood, and improper sleep in adults with MetS. These differences are probably due to the methods used to measure inflammation or psychiatric symptoms.

In this study, high serum hs-CRP levels were associated with an increased incidence of suicidal ideation in adults with MetS. This is consistent with several studies showing that inflammation promotes suicidal ideation and that suicidal ideation also may promote inflammation [10, 11, 18]. This suggests that suicidal ideation and inflammation may be bidirectional [10], and that altered activation of the HPA axis [18] and the tryptophan–kynurenine pathway may be among the biological mechanisms linking suicidal ideation with inflammation [10, 51].

The strength of this study is its use of data from a nationally representative survey of Korean adults and its status as the first study to investigate associations involving inflammation, quality of life, and mental health symptoms in Korean adults with MetS. However, this study had several limitations. First, the design of this study was cross-sectional. Second, we used only serum hs-CRP levels to assess inflammation. Third, we could not use validated tools to assess the mental health of Korean adults because such instruments were not used in the KNHANES.

In conclusion, high serum hs-CRP levels in adults with MetS were associated with mobility problems, suicidal ideation, and lower overall quality of life. These findings suggest that the elevated inflammatory status in MetS is associated with mental health problems and decreased quality of life. Further prospective studies are needed to assess the impact of inflammation on the quality of life and mental health of patients with MetS.

## Abbreviations

MetS: metabolic syndrome
hs-CRP: high-sensitivity C-reactive protein
EQ-5D: EuroQol 5-dimension
HDL: high-density lipoprotein
IL-6: interleukin-6
TNF: tumor necrosis factor
HPA: hypothalamus–pituitary–adrenal
KNHANES: the Korean National Health and Nutrition Examination Survey
KCDC: the Korea Center for Disease Control and Prevention
NCEP-ATP III: the National Cholesterol Education Program Adult Treatment Panel III
BMI: body mass index
CI: confidence interval
ORs: odds ratios
